# How and why might interprofessional patient- and family-centered rounds improve outcomes among healthcare teams and hospitalized patients? A conceptual framework informed by scoping and narrative literature review methods

**DOI:** 10.3389/fmed.2023.1275480

**Published:** 2023-10-11

**Authors:** Erin Abu-Rish Blakeney, Jennifer Baird, Genevieve Beaird, Alisa Khan, Victoria M. Parente, Kevin D. O’Brien, Brenda K. Zierler, Kevin J. O’Leary, Bryan J. Weiner

**Affiliations:** ^1^Department of Biobehavioral Nursing and Health Informatics, School of Nursing, University of Washington, Seattle, WA, United States; ^2^Children's Hospital Los Angeles, Los Angeles, CA, United States; ^3^School of Nursing, Virginia Commonwealth University, Richmond, VA, United States; ^4^Department of Pediatrics, Harvard Medical School, Boston, MA, United States; ^5^Boston Children's Hospital, Boston, MA, United States; ^6^School of Medicine, Duke University, Durham, NC, United States; ^7^Department of Cardiology, School of Medicine, University of Washington, Seattle, WA, United States; ^8^Feinberg School of Medicine, Northwestern University, Chicago, IL, United States; ^9^Department of Global Health, School of Public Health, University of Washington, Seattle, WA, United States

**Keywords:** interprofessional, patient- and family-centered care, hospital, rounds, communication, safety, team-based care, routines

## Abstract

Poor communication within healthcare contributes to inefficiencies, medical errors, conflict, and other adverse outcomes. A promising model to improve outcomes resulting from poor communication in the inpatient hospital setting is Interprofessional Patient- and Family-Centered rounds (IPFCR). IPFCR brings two or more health professions together with hospitalized patients and families as part of a consistent, team-based routine to share information and collaboratively arrive at a daily plan of care. A growing body of literature focuses on implementation and outcomes of IPFCR to improve healthcare quality and team and patient outcomes. Most studies report positive changes following IPFCR implementation. However, conceptual frameworks and theoretical models are lacking in the IPFCR literature and represent a major gap that needs to be addressed to move this field forward. The purpose of this two-part review is to propose a conceptual framework of how IPFCR works. The goal is to articulate a framework that can be tested in subsequent research studies. Published IPFCR literature and relevant theories and frameworks were examined and synthesized to explore how IPFCR works, to situate IPFCR in relation to existing models and frameworks, and to postulate core components and underlying causal mechanisms. A preliminary, context-specific, conceptual framework is proposed illustrating interrelationships between four core components of IPFCR (interprofessional approach, intentional patient and family engagement, rounding structure, shared development of a daily care plan), improvements in communication, and better outcomes.

## Introduction

Gaps in patient safety exist in inpatient hospital care. Research to improve safety for hospitalized patients has focused primarily on technological reporting and interventions. Medical errors have been recognized as the third leading cause of death in the United States for nearly a decade, and 40 % of hospital admissions are thought to include an adverse event or error (1–4). Further, errors and harms occur disproportionately for some groups (5–7). For example, a recent systematic review by Chauhan et al. (7), found higher rates of medication errors and hospital acquired infections among patients from ethnic minority backgrounds and those that use a language other than English for healthcare. A major driver of these challenges is thought to be poor communication within and between healthcare teams (1–3, 8). Health policy makers have repeatedly called for interventions to improve communication in practice (2, 8).

New and innovative approaches to improving safety, equity, and patient- and family-centeredness of hospital care need to be developed and studied to identify evidence-informed interventions that can be implemented into practice. One possible direction is identification and implementation of models, processes, or routines that change how care is organized and delivered. Team-based or interprofessional care models, including a model of daily inpatient care planning rounds known as interprofessional patient- and family-centered rounds (IPFCR), offer a promising approach. IPFCR brings two or more health professions together with patients and families as part of a consistent, team-based routine to share information and collaboratively arrive at a daily plan of care in inpatient hospital settings.

Rounds occur for almost every single patient, almost every single day in almost every hospital in the United States. Within this daily routine, however, formats vary widely and there are multiple overlapping and sometimes competing perspectives on the purpose of hospital rounds (e.g., patient care, updating families, formulating plans, teaching trainees) (9). Rounding as a care process is historically varied in terms of who is present, who contributes, when it occurs, where it occurs, what is discussed, and what decisions or outcomes are expected as a result (10–12). This combination of ubiquity and high variability is what makes rounds an opportune focus for study and improvement efforts. Recent growth in IPFCR interventions also suggests timeliness and front-line interest.

Despite a growing body of literature reporting promising results from IPFCR interventions, descriptions are highly variable and predominantly atheoretical (13). Further, evaluations of IPFCR across settings and populations have not been synthesized. The *objective* for this review is to begin to close these gaps by synthesizing existing IPFCR literature within the context of relevant theories and frameworks from related fields. The *overarching goal* is to offer a preliminary conceptual framework that guides the use of IPFCR and how it might be expected to lead to improvements in care and outcomes. This will provide a foundation upon which more generalizable knowledge can be built.

## Review scope and approach

This review was carried out in three parts. First, manuscripts included in a recently published systematic scoping review, which described new implementations of IPFCR models, were qualitatively reviewed and analyzed to identify themes (13). Second, a focused literature search was conducted to explore existing theories and frameworks that could inform a context-specific IPFCR conceptual framework. Finally, an IPFCR conceptual framework is proposed that includes four core components and illustrates relationships between these components and improvements in communication that have the potential to lead to safer, more equitable, and more patient- and family-centered care.

### Part 1: Systematic scoping review to identify common themes in published IPFCR literature

As a first step, articles included in a recent scoping review led by the first author of this paper were revisited to qualitatively explore whether and how they described how implementation of IPFCR leads to improved team and/or patient outcomes (13). The review methods, including search terms and inclusion/exclusion criteria, are described in detail elsewhere (13) and used a systematic approach to search PubMed, CINAHL, PsycINFO, and EMBASE to identify manuscripts describing new implementations of IPFCR models in pediatric and adult settings. The review identified 74 studies dating from 1988 and a recent steepening growth trajectory with 5 to 13 articles published each year from 2014 to 2020 (13). It described trends and gaps in the IPFCR literature and identified predominantly positive or neutral impacts following IPFCR implementation across an array of outcomes—including team communication, length of stay, and safety (13).

Of the 74 studies included in the scoping review, 42 (53.2%) described, explicitly or implicitly, how they expected implementation of IPFCR to improve team and/or patient outcomes as well as how these outcomes are interrelated (see Appendix A). Whether or not an article addressed this topic was determined during REDCap-based data abstraction and was confirmed by the lead author. Data abstractors answered two questions during full text review that provided the basis for the qualitative analysis described below. The first, a yes/no question, asked “does the study describe a tested or hypothesized ‘mechanism of action’ for the rounding model and/or its implementation?” The second was short answer item: “If yes, please describe and be sure to include whether the description is about the rounding model itself or the implementation of the rounding model.”

The lead author of this manuscript iteratively reviewed and made notes while reading the short answer items and cross-referenced them with the original manuscripts to identify implicit and explicit ways in which the authors expected implementation of IPFCR to improve team and patient outcomes. A qualitative synthesis of these descriptions pointed to three common themes:

*Theme 1*: Implementing a standardized model or approach to rounding provides an explicit framework for care planning and delivery.

Utilizing a consistent approach was described as helping to decrease variation (14), to increase use of evidence-based care and checklists by way of shared accountability and/or nudging (15, 16), and to make teaming among frontline care professionals possible by routinizing/synchronizing times and places for them to coordinate with each other (17, 18).

*Theme 2*: Engaging patients, families, and interprofessional team members is made possible when a standardized approach becomes routine.

As described in several articles, shifting rounds to the patient bedside is a key strategy to increase and sustain involvement of patients and family members in information exchange and decision-making during care planning, which can help improve patient- and family-centeredness of care, hospital experience, and mitigate safety errors and risks (19–24).

*Theme 3*: Providing regular opportunities for communication among interdependent care team members from multiple professions improves team relationships and contributes to the development of a shared understanding and agreement of patient care plans and goals (13, 18).

The result of improvements in communication and development of a shared mental model are then thought to improve the safety and quality of care by decreasing omissions or duplication of needed care, helping to prevent or decrease medical errors, and enhancing the hospital experience (14, 23–26). Improvements in communication are also described as improving job satisfaction among care team members (18, 20).

These themes suggest a shared belief among study authors that implementation of an IPFCR model can improve team and patient outcomes. Conversely, ineffective communication and unavailability of team members can negatively influence care and outcomes, create barriers to teamwork associated with adverse events, decrease satisfaction among care team members, patients, and families, and increase costs (25, 26).

### Part 2: Existing theories and conceptual frameworks to support or contradict emergent themes

Following identification of common themes in the first phase of this review, we conducted a focused literature search using a narrative review approach (27) to explore existing theories and conceptual frameworks to increase understanding of the emergent themes and inform a context-specific IPFCR conceptual framework.

In the above-described IPFCR scoping review, twenty-five studies (31.7%) cited a conceptual framework or theory supporting their work (13) (see Appendix A). Of those, the most commonly cited theories or frameworks originated from the fields of change management, quality, or systems improvement (*n* = 17, 68%) (28–32). A smaller number of studies, three each, referenced an interprofessional framework or a model of change framework. While each of the cited theories and frameworks provided useful framing for the studies in question, none were specific to rounds. Also absent were equity considerations within the existing frameworks.

This prompted additional literature review following a narrative approach (27) and subsequent identification of existing theories that focus on alternative models of rounds. Databases iteratively searched in this phase included PubMed, CINAHL, PsycINFO and EMBASE and utilized two primary search terms “rounds” and “theory” both individually and then combined (e.g., rounds and theory). For both terms, related concepts and key words were also searched (e.g., hospital rounds, physician rounds, nursing rounds; conceptual framework, theoretical framework, model). Abstracts and full text manuscripts were reviewed as they were identified and retained if they supported or contradicted emergent themes from Part 1. Described and synthesized below are the manuscripts and theories selected during this phase of searching and how they support or contradict emergent IPFCR themes identified above.

Two sets of papers were identified that focused specifically on uniprofessional models of rounds (e.g., physician-only or nurse-only models). In the first, Perversi et al. (33) focused on reasoning mechanisms in uniprofessional ward rounds used by physician teams to plan daily care. After observing 11 days of physician ward rounds for 94 individual patients, using a critical realist multiple case study approach, the authors identified several group reasoning mechanisms concerning sharing, agreeing, and recording information in the categories of information accumulation, sense-making and decision making to form a program theory of physician ward round reasoning. This paper provides compelling justification for the routine of daily care planning rounds to support information sharing and development of a shared mental model among participants. Notably absent from this model are patients, families, and other care team members (i.e., nurses, pharmacists, social workers), all of whom have information to share and whose life and daily work are impacted by the decisions made during these important rounding discussions. Further missing from this model is a consideration of how these approaches contribute to team, patient, and family outcomes.

The second set of papers, by Harris et al. (34) and Sims et al. (35), focused on a uniprofessional nurse rounding model. These studies used a realist evaluation and realist synthesis approach to studying “intentional rounding” by nurses during handoffs between shifts to improve engagement between nurses and patients (34, 35). The authors synthesized the results of a three-stage literature search and stakeholder consultation to identify eight *a priori* program theories to further understand what works in intentional rounding, for whom, in what circumstances, and why. The eight propositions that they identified were: (1) when implemented in a comprehensive and consistent way, intentional rounding improves healthcare quality and satisfaction; (2) embedding intentional rounding into daily routine practice gives nurses ‘allocated time to care’; (3) documenting intentional rounding increases accountability and raises fundamental standards of care; (4) when workload and staffing levels permit, more frequent nurse–patient contact improves relationships and increases awareness of patient comfort and safety needs; (5) increasing time when nurses are in direct vicinity of patients promotes vigilance, provides reassurance, and reduces potential harms; (6) more frequent nurse–patient contact enables nurses to anticipate patient needs and take pre-emptive action; (7) intentional rounding documentation facilitates teamwork and communication; and (8) intentional rounding empowers patients to ask for what they need to maintain their comfort and well-being. Thus Harris et al. (34) and Sims et al. (35) contribute to our understanding of the types of interactions and activities that occur on twice- or thrice-daily nursing handoff rounds (at each nursing shift transition) and how they might influence overall care and outcomes. While this model describes increased nurse–patient contact as improving relationships and increasing awareness and vigilance among nurses it does not address the perspective of patients or families in this process or explicitly engage them. However, like the physician-focused study of Perversi et al. (33), the Harris (34) and Sims (35) studies omit key partners in the process of care by focusing on nurses, as opposed to the interprofessional care team (33, 34).

In contrast to the uniprofessional nurse- or physician- focused rounding models described above, Kydonaki et al. (36) applied an integrative approach to their review of 15 articles to explore family involvement in ward rounds for adult ICU patients. They summarize their findings in a 3-part framework of “involvement of family members in rounds.” This is broken down into three concepts: (1) interactions and communication during rounds, (2) organization of rounds, and (3) ICU culture. Each of the three concepts is further broken down into two or three sub-concepts. Interactions and communication during rounds is divided into two sub-concepts of: (1) increase of situational awareness and involvement in decision making and (2) advancing emotional experience (e.g., satisfaction, experience). Organization of rounds is divided into: (1) structure and process of rounds, (2) use of communication tools, and (3) roles in rounds. ICU culture is broken down into (1) value in family-centered rounds and (2) barriers in family-centered rounds. The authors identify positive attitudes of family members and patients toward involvement in family rounds, but the review does not provide quantitative data on other patient- and family-centered outcomes, such as mental health outcomes, nor qualitative data to understand the barriers, processes, and facilitators to implementing family-centered rounds in ICUs. Kydonaki et al.’s review included both uniprofessional and interprofessional rounding approaches so long as the approaches focused on engaging family members in rounds. One notable finding they report is a mismatch between healthcare professionals’ perceptions of family member desire to participate in rounds (they perceived 38% of family members as wanting to participate) and expressed desire of family members to participate in rounds (97% indicated that they would like to participate) in the same setting (37). This review focused more on what was done and what was found in the included articles in terms of family engagement in rounds and less on mechanisms of how rounds worked or why they did or did not meet the needs of patients, family members, or other care team members.

Similar to Kydonaki et al., Reeves et al. used a comparative ethnographic approach. (observations, interviews, and document review) to explore the culture of interprofessional collaboration and family member involvement in 8 ICUs in North America. While not focused explicitly on rounds, rounds were observed and the researchers utilized a 4-domain interprofessional conceptual framework to guide their data collection and analysis. Domains include (1) relational factors (i.e., how power, hierarchy, and leadership influence relationships), (2) processual factors (i.e., time, space, and task complexity as processes of collaboration), (3) organizational factors (i.e., impacts of local institutional structures and management processes), and (4) contextual factors (broader cultural, political, social, and economic issues as they influence interprofessional collaborative practice) (38, 39). The authors found that interprofessional collaboration occurred most commonly during emergent situations and less commonly during more routine activities, such as rounds or handoff activities, which the authors found to be predominantly uniprofessional and heavily influenced by historic professional hierarchies. They also found that family members played important roles in communication and care both for the patient as well as *within and between different professions*. Similar to Kydonaki et al. (36), the framework and findings described by these authors are illustrative of what was happening in ICUs as it related to interprofessional collaboration and family member involvement. However, they do not shed light on the mechanistic aspects of rounds’ cognition and dynamic interaction described in the two uniprofessional papers.

### Part 3: Integration of scoping and narrative reviews to inform a preliminary IPFCR model and theory

**Figure 1:**
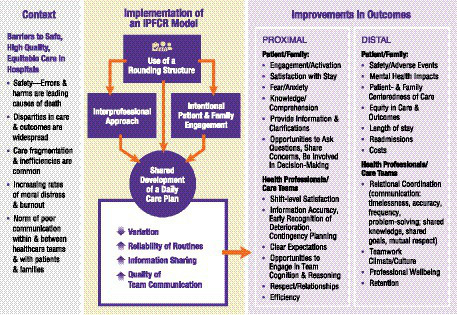
Preliminary context-specific conceptual framework linking core components of an interprofessional patient- and family-centered rounding (IPFCR) model to improvements in team and patient outcomes.

Based on a synthesis of existing literature and relevant theories in Parts 1 and 2 above, we propose four core components (Table 1) and a preliminary context-specific IPFCR conceptual framework (Figure 1).

**Table 1:** IPFCR model definition and core components.

**Definition:** Rounding model that brings two or more health professions together with patients and families as part of a consistent team-based routine to share information and collaboratively arrive at a daily plan of care	
**Component**	**Description**
1. Interprofessional collaboration	Rounding as an interprofessional team with representatives of multiple professions/disciplines (e.g., nurses and physicians).
2. Intentional patient and family engagement	Performing rounds at the bedside (if permitted by patient and family) and inviting information and perspective sharing and questions during care planning & decision making.
3. Rounding structure	Utilizing a predetermined process for speaking roles, presentation order, and suggested content (e.g., vitals, assessment, plan).
4. Shared development of a daily care plan	Review of patient data during rounds results in the formulation of a plan of care for the day and beyond with input from the entire team (including patients and family members/caregivers).

Each of the four core components defined—(1) interprofessional collaboration, (2) intentional patient and family engagement, (3) rounding structure, (4) development of a daily shared care plan—are distinct but interdependent and each is hypothesized to be necessary to achieve safe, high quality, equitable hospital care and ensure intervention effectiveness (40). The underlying theory is that introducing structured routines like IPFCR can help foster “high-reliability” practices in healthcare organizations reducing variations in care through standardized approaches and improved communication, thereby leading to better outcomes (41, 42). This theory is consistent with the themes identified in Part 1 of this review and supported by a growing body of research that associates IPFCR with improvements in team and patient outcomes (13, 43–47).

Figure 1 illustrates proposed connections between the four core components, as well as proximal and distal outcomes. The draft visual model was developed iteratively using the structure-process-outcome models in Parts 1 and 2 of this review, and a recently published toolkit: “Building Implementation Roadmaps: A Toolkit for Creating Causal Pathway Diagrams” (48, 49).

The draft model, which moves from left to right, begins with acknowledging the many long-standing challenges and barriers to safe, high quality, equitable care in the United States healthcare. Next, interrelationships between the four proposed core components of an IPFCR model are portrayed, illustrating how the use of a rounding structure provides a supportive structure for interprofessional care team members to come together with patients and families to develop a shared care plan. It is hypothesized that the result of implementing the four IPFCR core components increases in the reliability of rounding routines that support information sharing and better team communication. Together, we hypothesize that these activities lead to improvements in both proximal and distal outcomes for patients, families, health professionals, and the overall care team.

## Discussion

This review uses literature review and thematic analysis to propose a conceptual framework of IPFCR that highlights the importance of interprofessional collaboration, patient and family engagement, structure, and development of a daily shared care plan. This framework will enable future studies to clarify whether similar-sounding models described in the literature are in fact, similar, in both form and function. Additional research is necessary, because it is unclear from the current literature what is essential or core to an optimal IPFCR model and how an IPFCR model might improve team and patient outcomes that to make care safer, more equitable, and more patient- and family-centered.

As a process that introduces principles of high reliability, IPFCR models provide an environment for team cognition as described in phase 2 of this review (41, 50). Utilizing high reliability as a foundational concept provides important perspective, as it includes an appreciation that patient care is complex and complexity is better addressed when an interprofessional care team, including patients and families, is involved. Principles of high reliability also guide users to avoid harmful oversimplification, unconsidered variation, and inequities and the proposed model helps to account for this complexity and current variability in care processes.

Another body of research that supports the potential impacts of IPFCR models on patient care is around organizational routines. Across many sectors, routines are used to help coordinate processes and reduce uncertainty. When IPFCR models are implemented consistently, they serve as a structuring device of collaboration and organizational learning (51). As effective communication plays such a critical part in improving outcomes, there is inherent value in increased focus on the routine structures designed for information sharing across professions, patients, and families (52). As for future research on IPFCR, using guidance from existing research on organizational routines and from the implementation science literature may be helpful for establishing consistency in reporting important details of the routine (i.e., who is involved, leadership, location, any variability from established guidelines, etc.). Sharing these details will allow scholars to compare findings more accurately across studies (53, 54).

### Limitations

This manuscript review delved into multiple areas of literature. Because Part 2 presented a focused, rather than formal systematic, search there is possibility of bias through the omitting or limiting of relevant literature in that section.

### Conclusion

The proposed conceptual framework offers a synthesis of practice-based evidence and theory about how and why rounds “work.” Inherent in this framework is an assumption that rounds can work even better when they use a standardized approach that is more inclusive of interprofessional care team members, patients, and families. By defining this IPFCR framework in terms of core components informed by theory, an opportunity for more rigorous future study is created. Studies using an explicitly defined conceptual framework of IPFCR are essential to determining whether it is important to optimize, scale, and spread IPFCR models (54).

## Author contributions

EB: Conceptualization, Data curation, Formal analysis, Funding acquisition, Investigation, Methodology, Project administration, Resources, Software, Supervision, Validation, Visualization, Writing – original draft, Writing – review & editing. JB: Writing – review & editing. GB: Writing – review & editing. AK: Writing – review & editing. VP: Writing – review & editing. KO’B: Writing – review & editing. BZ: Writing – review & editing. KO’L: Visualization, Writing – review & editing. BW: Supervision, Visualization, Writing – review & editing.
