# Supplementary material for: How and why might interprofessional patient- and family-centered rounds improve outcomes among healthcare teams and hospitalized patients? A conceptual framework informed by scoping and narrative literature review methods
Source: Front Med (Lausanne). 2023 Oct 11;10:1275480. doi: 10.3389/fmed.2023.1275480 (PMC10598853; doi:10.3389/fmed.2023.1275480)
Supplement: Supplementary file 1 [file Table_1.DOCX]

**Appendix A.** Abbreviated details of studies citing theories or describing how they expected interprofessional rounds to lead to improvements in team and/or patient outcomes in chronological order (1988-June 30, 2020). (n=42)

| First Author (Year)⸺Article Title | Author references to guiding theories and/or hypothesizes connecting IPFCR implementation to improved team and/or patient outcomes |
| --- | --- |
| Lewis et al. (1988)⸺Patient, parent, and physician perspectives on pediatric oncology rounds | Authors hypothesize that rounding model can improve communication, trust, and relationships between patients/parents and physicians. |
| Young et al. (1998)⸺The impact of a multidisciplinary approach on caring for ventilator-dependent patients | Continuous Quality Improvement/ Collaborative Care Model; Authors describe purpose as: “"designed to determine whether a collaborative care model, focused on reducing variation and promoting collaborative care for ventilator-dependent patients (VDP), leads to improved medical outcomes and decreased in length of stay and costs.” |
| Dodek & Raboud (2003)⸺Explicit approach to rounds in an ICU improves communication and satisfaction of providers | Authors hypothesized that an explicit approach to clinical and educational responsibilities and to reporting assessments and plans during bedside rounds in an ICU would improve communication and satisfaction of health care providers. |
| Fertleman et al. (2005)⸺Improving medication management for patients: the effect of a pharmacist on post-admission ward rounds | Authors hypothesize that “if the pharmacist was present when prescribing decisions are made, there would be greater opportunities to review current medication, reduce polypharmacy, and reduce medication related risks and costs. In addition, recommendations for commencing appropriate drugs for new conditions may be made." |
| Monaghan et al. (2005)⸺Improving patient and carer communication, multidisciplinary team working and goal-setting in stroke rehabilitation | UK National Service Framework |
| Bonello et al. (2008)⸺An intensive care unit quality improvement collaborative in nine department of veterans affairs hospitals: reducing ventilator associated pneumonia and catheter-related bloodstream infection rates | Authors reference IOM reports and hypothesize that having a pharmacist on the rounding team should reduce adverse events. |
| Latta et al. (2008)⸺Parental responses to involvement in rounds on a pediatric inpatient unit at a teaching hospital: A qualitative study | Family Centered Care—from discussion section “"In theory, participation of parents during interdisciplinary rounds should improve communication and, thereby, improve patient safety and quality of care. Improved communication should also enhance prevention of omissions or duplication of needed care, thus reducing cost and length of stay." |
| Jacobowski et al. (2010)⸺Communication in critical care: Family rounds in the intensive care unit | Authors hypothesize that family rounds provide early/consistent communication to better facilitate end of life planning when needed. |
| Rappaport et al. (2010)⸺Implementing family-centered rounds: pediatric residents’ perceptions | Patient Centered Medicine (originally described by Balint in the 1960’s)/ Family Centered Rounds |
| Licata et al. (2013)⸺A foundation for patient safety: Phase I implementation of interdisciplinary bedside rounds in the pediatric intensive care unit | Patient Centered Care; references IOM reports |
| Saint et al. (2013)⸺An academic hospitalist model to improve healthcare worker communication and learner education: results from a quasi-experimental study at a Veterans Affairs Medical Center | Circle of Concern Rounds described by Suzanne Gordon |
| Oshimura et al. (2014)⸺Family-centered rounding: Can it impact the time of discharge and time of completion of studies at an academic children’s hospital? | Authors state that theoretically, “nursing participation may assist the physician team in anticipating the patient's medical and social needs earlier in the hospital stay to result in a more timely hospital discharge.” |
| Palokas et al. (2014)⸺An interactive evaluation of patient/family centered rounds on pediatric inpatient units | PDSA |
| Seigel et al. (2014)⸺Successful implementation of standardized multidisciplinary bedside rounds, including daily goals, in a pediatric ICU | PDSA Cycles of Improvement |
| Southwick et al. (2014)⸺Applying athletic principles to medical rounds to improve teaching and patient care | Toyota Production System; Athletic Training Principles |
| Wrobleski et al. (2014)⸺Discharge planning rounds to the bedside: A patient- and family-centered approach | Quality Health Outcomes Framework (Mitchell, et al. 1998) (page 112 in reference to framework states that "this framework suggests system, provider, patient, and family characteristics influence intervention outcomes... including effective communication and unavailability of hospital or community practitioners |
| Stein et al. (2015)⸺Reorganizing a hospital ward as an accountable care unit | High Value Care; Clinical Microsystems. States that: “modern hospital units could benefit from having a standard care model that synchronizes frontline professionals into teams routinely coordinating and progressing a shared plan of care") |
| Tripathi et al. (2015)⸺Implementation of patient-centered bedside rounds in the pediatric intensive care unit | PDSA |
| Allen et al. (2016)⸺A novel method of optimizing patient- and family-centered care in the ICU | Shared decision making |
| Braus et al. (2016)⸺Prospective study of a proactive palliative care rounding intervention in a medical ICU | Nudges (palliative care specialist joined ICU IPFCR based on EHR based trigger tool and provided recommendations to better meet PC needs of patients and families) |
| Gustafson et al. (2016)⸺Effect of parent presence during multidisciplinary rounds on NICU-related parental stress | Authors hypothesize that integration of parent’s into interprofessional NICU rounds would decrease parental stress by enhancing understanding of infant’s condition, plan of care, and facilitation communication with the team |
| Henkin et al. (2016)⸺Improving nurse-physician teamwork through interprofessional bedside rounding | Authors hypothesize: “involving nurses and physicians in rounds can help "create a shared mental model and increase collaborative activities" |
| Justice et al. (2016)⸺Improving communication during cardiac ICU multidisciplinary rounds through visual display of patient daily goals | Improvement Science Model with the hypothesis being that improving communication efficacy will help ensure understanding of/agreement on patient goals |
| Okere et al. (2016)⸺Comparison of a pharmacist-hospitalist collaborative model of inpatient care with multidisciplinary rounds in achieving quality measures | Authors hypothesize that pharmacist-hospitalist collaborative model of care will decrease LOS and readmissions |
| O'Leary et al. (2016)⸺Effect of patient-centred bedside rounds on hospitalised patients’ decision control, activation and satisfaction with care | Authors hypothesize that conducting rounds at the bedside would improve patient participation in decision-making and satisfaction with care. |
| Young et al. (2016)⸺Impact of altered medication administration time on interdisciplinary bedside rounds on academic medical ward | Authors describe parallel asynchronous workflow leading to adverse events, dissatisfaction, increased costs; hypothesized that an IPCC (Team-based healthcare) approach could lead to improvements |
| Huang et al. (2017)⸺All together now: Impact of a regionalization and bedside rounding initiative on the efficiency and inclusiveness of clinical rounds | Authors hypothesize that regionalization of care teams and encouragement of rounding would affect time measures of rounding and participation |
| McGrath et al. (2017)⸺Evaluating the quality improvement impact of the Global Tracheostomy Collaborative in four diverse NHS hospitals | PDSA Cycles |
| Thorne et al. (2017)⸺Co-producing interprofessional round work: designing spaces for patient partnership | Model of Clinical Microsystems and Social-Material Practice Theory Perspective |
| Abu-Rish Blakeney et al. (2018)⸺Purposeful interprofessional team intervention improves relational coordination among advanced heart failure care teams | Relational Model of Organizational Change |
| Baik & Zierler (2018)⸺RN job satisfaction and retention after an interprofessional team intervention | Relational Model of Organizational Change |
| Cody et al. (2018)⸺Making a connection: Family experiences with bedside rounds in the intensive care unit | Family Management Style Framework |
| Khan et al. (2018)⸺Patient safety after implementation of a coproduced family centered communication programme: multicenter before and after intervention study | Authors hypothesize that improved communication amongst all would improve shared understanding and help prevent medical errors as well as improve hospital experience. |
| Li et al. (2018)⸺Interprofessional Teamwork Innovation Model (ITIM) to promote communication and patient-centred, coordinated care | Authors reference IOM reports, systems theory and structure, process, outcomes approach in discussion section (not up front) and hypothesize that working in IP teams as the best way to achieve the Quadruple Aim |
| Mork et al. (2018)⸺Using Kotter’s Change Framework to implement and sustain multiple complementary ICU initiatives | Kotter model of change |
| Gormley et al. (2019)⸺Impact of nurse-led interprofessional rounding on patient experience | PDSA Model |
| Lopez et al. (2019)⸺Impacting satisfaction, learning, and efficiency through structured interdisciplinary rounding in a pediatric intensive care unit: A quality improvement project | Authors hypothesize that shifting to an structured interprofessional rounding model will improve communication, thereby leading to improvement in many other aspects of performance—including unit workflow, family and staff satisfaction, and decreases in LOS. States, “states "a key benefit of IBR is fostering effective, accurate, and timely communication by bringing together team members with the patient and family at the same time and place. It promotes shared situational awareness and is essential to delivering high-quality care." |
| Opper et al. (2019)⸺Effects of implementing a health team communication redesign on hospital readmissions within 30 days | Meleis’ Transitions Theory; authors describe IBR rounds as a “mechanism for direct communication between health care members and patients and families to assure common goals, proactive planning, and consistent information exchange.” (p. 122) |
| Austin et al. (2020)⸺Evaluation of a nurse practitioner-led project to improve communication and collaboration in the acute care setting | AIDET Format (Acknowledge-Introduce-Duration-Explanation-Thank You) |
| Kang et al. (2020)⸺State anxiety, uncertainty in illness, and needs of family members of critically ill patients and their experiences with family-centered multidisciplinary rounds: A mixed methods study | Authors hypothesize that family-centered multidisciplinary rounds could alleviate anxiety and uncertainty in illness and meet needs for critically ill patients’ families. |
| Redley et al. (2020)⸺Mixed methods quality evaluation of structured interprofessional medical ward rounds | Authors hypothesize that standardizing the structure and content of interdisciplinary ward rounds is proposed to promote patient safety and patient-centered collaboration. States that, structured interdisciplinary bedside rounding (SIDR) “ uses evidence-based principles to guide clinician behaviors and processes during bedside ward rounds to deliver improvements in: (i) patient engagement and participation, (ii) patient-centered interdisciplinary teamwork; (iii) streamlined processes for service efficiency; (iv) use of standard processes and checklists to detect and mitigate safety risks |
| Sunkara et al. (2020)⸺Impact of structured interdisciplinary bedside rounding on patient outcomes at a large academic medical center | Authors reference IHI’s proposed general guidelines that support the use of IDR and interdisciplinary team-based care as a “mechanism for identifying patient safety risks and determining daily goals.” |
